# FRAILTY AND OSTEOARTHRITIS IN OLDER CHILEANS

**DOI:** 10.1093/geroni/igac059.2119

**Published:** 2022-12-20

**Authors:** Cecilia Albala, Carlos Marquez, Barbara Angel, Rodrigo Saguez, Lydia Lera

**Affiliations:** INTA, Universidad de Chile, Santiago de Chile, Region Metropolitana, Chile; INTA, Universidad de chile, Macul, Region Metropolitana, Chile; INTA, Universidad de chile, Santiago de Chile, Region Metropolitana, Chile; INTA, Universidad de chile, Santiago de Chile, Region Metropolitana, Chile; INTA, Universidad de chile, Santiago de Chile, Region Metropolitana, Chile

## Abstract

The adverse consequences on health of frailty, make it early diagnostic very important. The coexistence of frailty with osteoarthritis, a frequent condition especially in older women, has been less studied Objective. to study the association between frailty and self-reported osteoarthritis.

Methods: Cross-sectional study in994 people65 years and older (72.1%women, mean age72y±6.7) from the Alexandros cohort, designed to study disability associated with obesity in community-dwelling people60y and older living in Santiago/Chile. The frailty phenotype was defined as having ≥3 from the5 following criteria: weak handgrip dynamometry, unintentional weight loss, fatigue/exhaustion, five chair-stands/slow walking speed and low physical activity. Self-reported osteoarthritis was registered.We found a prevalence of osteoarthritis was much higher in women than in men (49.4% vs22.9%, p<0.01) Osteoarthritis was present in46.5% of frail people and30% of the robust ones (p=0.01). Frailty was present in53.3% of women with OA in and11.8% of men with OA. The crude OR for the association of frailty with Osteoarthritis was significant only for women (OR=1.43;95% CI:1.06-1.93). After sex and age adjusted logistic regression for frailty in Ostroarthritis, the OR for frailty was OR=1,39;95%IC (1,04-1,85), p=0,025, but in women with osteoarthritis the adjusted OR for frailty increase to OR=11.98 (5.637-13.23)Considering the severe consequences of frailty over health, the high burden of Osteoarthritis, its high frequency in women, and the strength of the association between both conditions, the screening for frailty is highly recommended In older women with Osteoarthritis.

